# Optimised hybrid late fusion deep learning model for cashew disease classification

**DOI:** 10.3389/fpls.2026.1815033

**Published:** 2026-05-22

**Authors:** Meenakshi K, Suresh Sankaranarayanan

**Affiliations:** 1 Department of Networking and Communications, School of Computing, SRM Institute of Science and Technology, Kattankulathur, Tamil Nadu, India; 2 Department of Computer Science, College of Computer Sciences and Information Technology, King Faisal University, Al-Ahsa, Saudi Arabia

**Keywords:** artificial intelligence, cashew plantation, deep learning, image classification, optimization

## Abstract

**Introduction:**

Cashew farms are highly vulnerable to the attack of pests and plant diseases, which leads to massive losses in production and economic value. To diagnose the cashew disease, recent studies have explored deep learning methods; however, the existing models often have poor representative features and low generaliz-ability to different situations.

**Methods:**

As part of these solutions, we suggest a hybrid late-fusion architecture (combining EfficientNetV2-M and MobileNetV3-S) to extract the features and train them with XGBoost and CatBoost to give the classification, using the BOHB optimisation to make the hyper-parameter choices. By combin-ing MobileNetV3-S and EfficientNetV2-M, we can benefit from fine-grained visual information and computer efficiency.

**Result and discussion:**

The proposed model was experimented on the CCMT dataset comprising Anthracnose, Gummosis, Leaf Miner, Red Rust, and healthy leaf samples, achieving classification accuracies of 90% and 93%, with reduced computation times of 0.20 seconds for the XGBoost classifier and 0.07 seconds for the CatBoost classifier. As our findings show, boosting-based classifiers and efficient backbone networks can be combined to identify cashew disease effectively and computationally less complex.

## 1 Introduction

Cashew nut is one of the four major dried fruits in the world and has been appreciated because of its high nutritional value (Liu et al., 2024). It is grown widely as a profitable cash crop in many areas of the world, such as tropical regions like southern China. The cashew tree is not just a food crop; it has several economic roles (Wang and Yang, 2002). FAO data shows that the cashew nut production in China dropped drastically in 2019, down to only 149 tonnes after 590 tonnes in 2009. The main cause of this decline can be researched to the spread of diseases and pests, the high environmental standards required to grow the crops, and the low level of awareness held by the farmers on how to handle the pests and diseases. The problems have complicated the cultivation process, as well as yield and quality, hence lowering the profitability of the cashew industry. Meanwhile, ITC Trade Map data shows that cashew demand in Asia is steadily rising. In China alone, imports of shelled cashews surged by over 70%, from 4,321 tonnes in 2017 to 30,688 tonnes in 2021.

**Figure 1:**
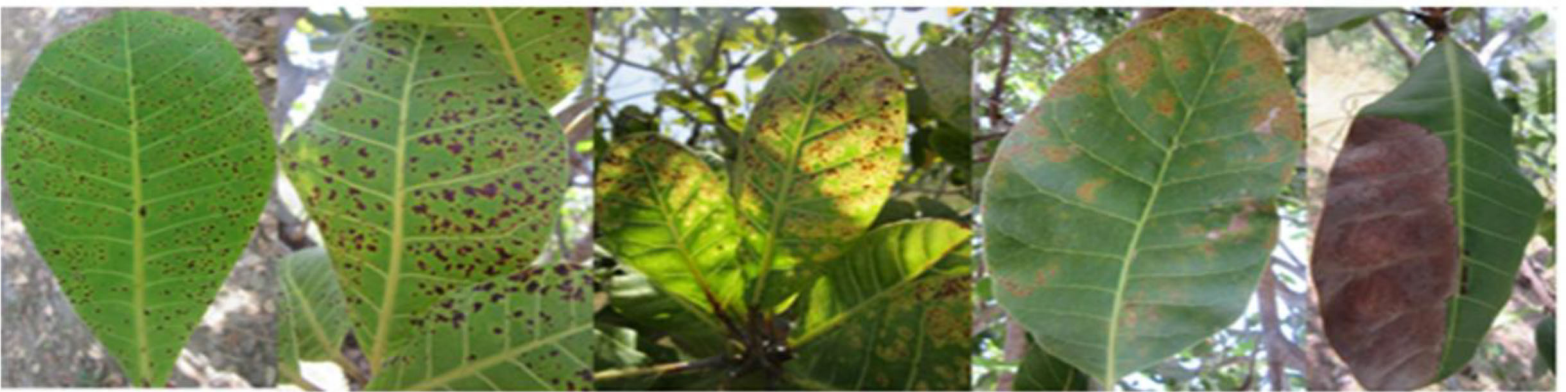
Foliar symptoms of Anthracnose.

**Figure 2:**
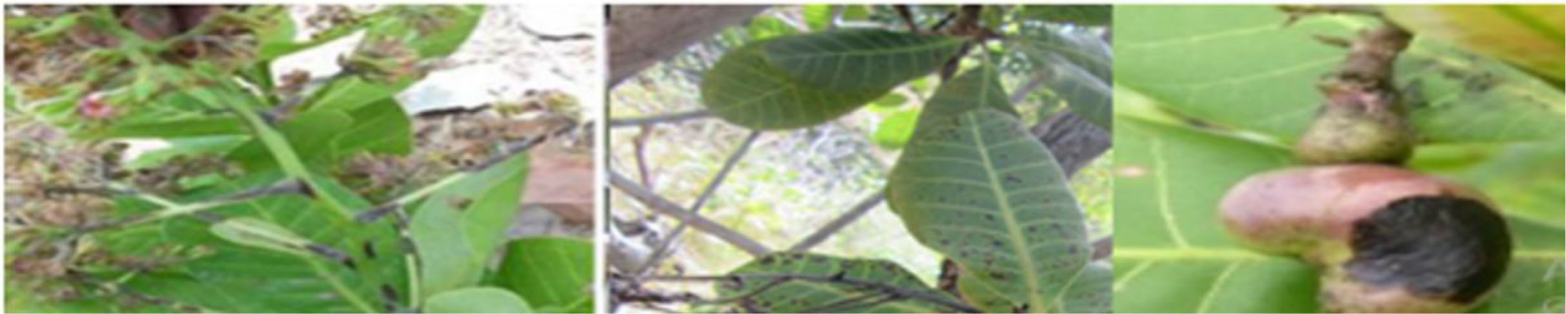
Symptoms of Anthracnose.

**Figure 3:**
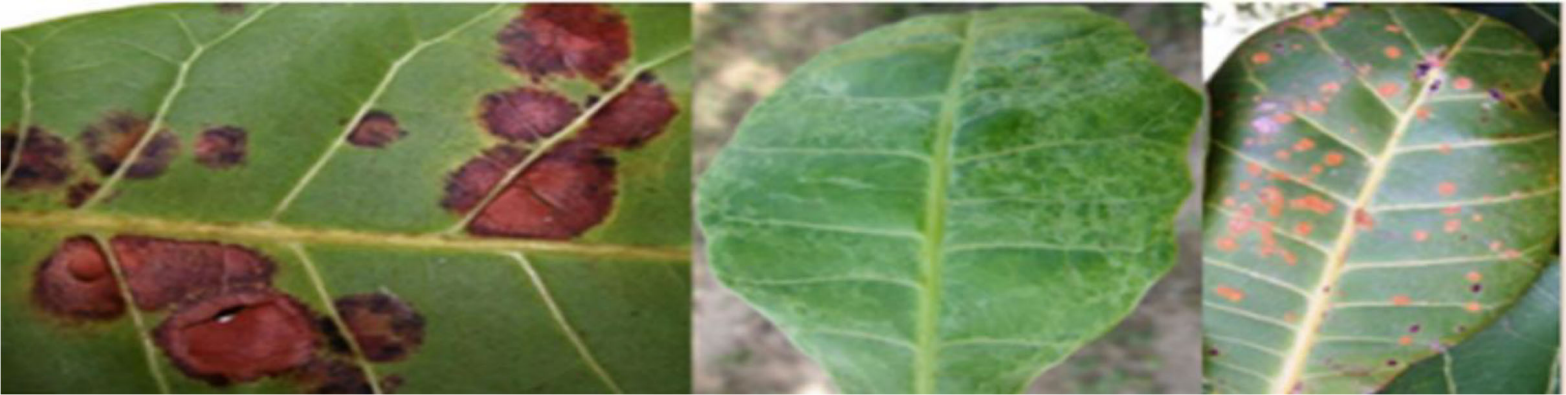
Pestalotia leaf spots.

More than twelve diseases have been reported to affect cashew worldwide. Among these, the major ones causing significant yield losses are grouped into three categories named Anthracnose, Pestalotia leaf spot, and gummosis. Current automated disease detection methods primarily rely on foliar symptom identification, even though some infections also affect the nuts and stems. **Figures 1**-**3** show the affected cashew leaves. In particular, the leaves of cashew nuts are particularly susceptible to pests and diseases during the growing process. Pest and disease-infected leaves may turn discolored and shed, which may reduce production and economic efficiency. Most places use manual methods for identifying diseases and pests, although this approach has numerous drawbacks (Agarwal et al., 2023).

Effective detection of pests and diseases in cashew cultivation requires specialized knowledge, which many farmers lack. The advancement of AI, particularly in Computer Vision techniques, offers effective solutions in automating plant disease and pest detection. Using this technology, cashew harvesting can be improved, yields can be enhanced, and specifically, the economic benefits of cashew production can be increased.

There are some challenges associated with traditional Machine Learning models of agricultural image disease classification, including the variability of data due to different lighting and backgrounds, which causes inconsistent feature extraction. Most models are relying on handcrafted features which are sensitive to variations and cannot easily generalize on controlled datasets to real-field conditions (Mohanty et al., 2016). Some of the limitations are class imbalance and overfitting due to a limited dataset (Demilie, 2024). Furthermore, black box models such as neural networks might not be trusted as interpretable by agricultural practitioners. In order to overcome these challenges, Deep Learning techniques, proper dataset and efficient generalized models will be needed (Ramanjot et al., 2023).

The current state of the art of deep learning is applied to disease classification in agricultural images. Recent works achieved with deep learning for agricultural image disease classification reveal certain challenges, such as high computational overhead, which limits their usability in low-resource environments and lightweight models that enhance inference speed but may compromise performance on complex datasets (Pacal et al., 2024). While the feature concatenation approach may help achieve high accuracy, it may introduce computational overhead, and ensemble models typically achieve better accuracy with a cost of latency and model size (Sambasivam et al., 2025). Some models show good performance for disease detection, but the problem of generalization arises from small datasets or heavily rely on controlled environment conditions for pest detection. Overall, most of the research papers state that the DL models produce promising results in agriculture, but these models face challenges such as computational expense, generalization, and deployment issues (Nyawose et al., 2025).

The main aim of the study is to design and develop a hybrid model incorporating the strong feature extraction properties of deep learning with the strong classification properties of boosting algorithms to accurately identify cashew leaf disease. The research aims to overcome the drawbacks present in existing machine learning (ML) and deep learning (DL) systems. Traditional ML methods heavily rely on manual feature engineering and are constrained in the ability to represent high-level features such as textures and colors of the plant leaves’ pictures. On the other hand, while being highly effective in automatic feature extraction, DL systems may suffer from overfitting, creation of redundant features, and decreased interpretability.

The key novelty introduced in this research is a new hybrid system combining the advantages of sophisticated deep learning networks and boosting techniques to improve the diagnosis of diseases on cashew leaves. In particular, the hybrid method uses EfficientNetV2-M and MobileNetV3-S for efficient feature extraction from the leaf images and helps to solve the problem of reliance on manual feature design in traditional ML techniques and possible problems with overfitting and feature redundancy in DL systems.

Additionally, by implementing the use of two classification models – XGBoost and CatBoost – the system can take advantage of their excellent classification performance, good behavior in imbalanced data sets, and anti-overfitting capabilities. The following contributions are proposed in this work.

- Build a hybrid deep learning model, which combines EfficientNet and MobileNet to increase feature extraction and boost the accuracy of classification of crop disease images and pest images.
- Enhance the classification framework using XGBoost and CatBoost on fused features while optimizing hyperparameters with Bayesian Optimised HyperBand (BOHB) to increase efficiency and improve classification accuracy.
- Validation of hybrid deep learning models using accuracy, precision, recall, F1-score, computation time to identify the most effective model for crop disease and pest classification.

**Figure 4:**
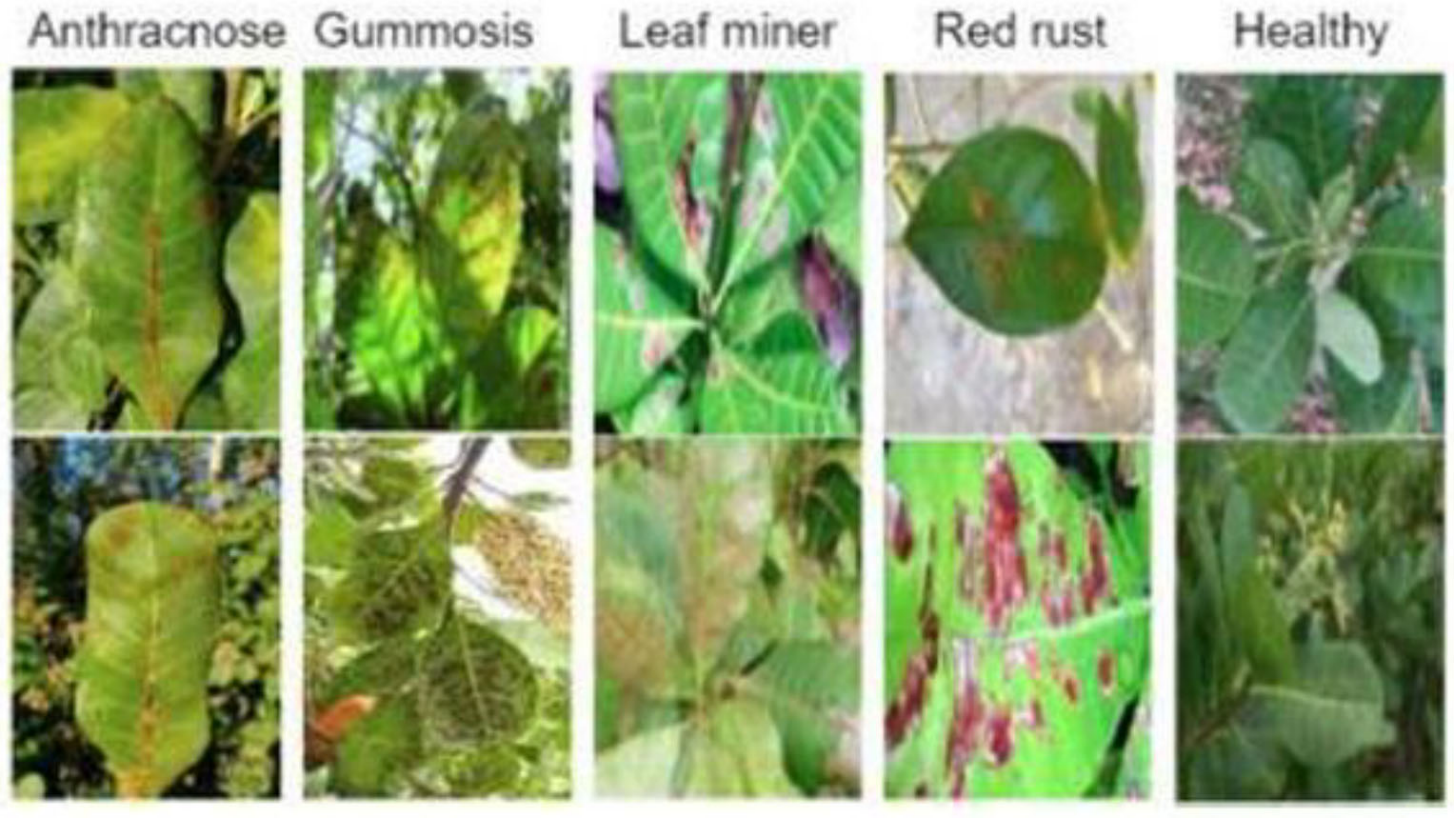
Sample images of the 5 classes.

The sample images of five classes of cashew leaves are shown in **Figure 4**. The rest parts of this manuscript are arranged as follows: Section II presents the literature survey on the identification of cashew plant diseases and the techniques. Section III describes the proposed work utilized in this study, Section IV presents the dataset and analysis of results, and lastly, Section V concludes the paper with future directions.

## 2 Related works

Several researchers have come up with various Machine Learning and deep learning algorithms to improve the precision of cashew leaf disease detection. These recent studies are analyzed here based on their methodologies, outcomes, and limitations, forming the base for improvements proposed in this research.

### 2.1 Machine learning-based methods

Mokhtar et al. (2015) suggested a novel image processing-based methodology in detecting tomato leaf disease with Gray-Level Co-occurrence Matrix (GLCM) features and Support Vector Machine (SVM) classification. They used the following method: they pre-processed in order to isolate the leaf region, then extracted the statistics features based on the texture using GLCM, and finally used SVM to differentiate between healthy and infected leaves. Still the model was efficient for binary classification and depended heavily on manually extracted texture features, which are sensitive to lighting, background, and imaging conditions. Kumari et al. proposed a model of leaf disease classification in which feature extraction was performed with the help of GLCM, and image segmentation was done with the help of K-means (Kumari et al., 2019). The final classification was performed by using a back-propagation neural networks.

Sarkar, and Pradhan (Kumar et al., 2019) developed a recommendation system for crop identification and pest control by integrating conventional feature extraction and classification techniques. The methodology of these studies processed the crop and pest images based on their color and texture characteristics and used Support Vector Machines (SVM) classifier, resulting in relatively high levels of accuracy compared to other classifiers. However, the effectiveness of this approach has been proven only in experimental settings using a small amount of data.

Another study done by Sudha and Kumaran (2023) examined the occurrence of anthracnose disease among cashew trees by combining K-means segmentation and a Random Forest classifier, which resulted in very high levels of accuracy on two datasets - 99.7% on the PDDB and 96.7% on the CCBBD dataset. These results are promising, but this research might face challenges related to overfitting and lack of diversity in the dataset.

Geetha et al. (2020) applied a k-Nearest Neighbors classifier for the identification of multiple tomato leaf diseases – such as bacterial spot, yellow leaf curl virus, and early blight, proving that simple instance-based algorithms can work well with small-scale data. However, the sensitivity of KNN algorithm to the scaling and variability in the data hinders its use in various scenarios. In another study, Deng et al. (2018) developed a machine learning technique for detecting pests in tea plants using a pattern descriptor inspired by the principles of human vision. They achieved significant success in classifying ten different types of pests with the help of SVM classifier. However, it should be noted that this technique is restricted by handcrafted features, which limit its ability to generalize in varying lighting and scales.

Finally, Rutkoski et al. (2012) provided an example of a predictive evaluation methodology based on multiple regression models, such as Random Forest regression, Bayesian LASSO, and RKHS regression for wheat pests. This technique allows identifying the impact of pests on yield using various statistical techniques. However, these models have been tested exclusively on dataset-based experiments and require generalization to be deployed in a real-world setting. **Table 1** lists the relevant studies in ML-based plant disease detection and classification.

**Table 1:** Summary of individual articles on machine learning based plant disease detection and classification.

Reference	Objective/focus	Methodology	Key outcomes	Drawbacks/limitations
Kumar et al. (2019)	Crop identification and pest control recommendation	Image-based feature extraction (color, texture) + SVM classifier	SVM achieved highest accuracy among tested models	Small dataset, tested in controlled conditions, limited real-field validation
Sudha & Kumaran (Sudha and Kumaran, 2023)	Cashew anthracnose disease detection	K-Means segmentation + Random Forest classifier	99.7% (PDDB) and 96.7% (CCBBD) accuracy	Possible overfitting, dataset not diverse, limited scalability
Geetha et al. (2020)	Tomato leaf disease detection	Feature extraction + KNN classifier	Successfully detected bacterial spot, yellow leaf curl virus, and early blight	Sensitive to data scaling and noise; limited robustness
Deng et al. (2018)	Tea pest classification	Local configuration pattern features + SVM classifier	Effective identification of 10 pest types inspired by human visual cues	Hand-crafted features sensitive to lighting, rotation, and scale changes
Rutkoski et al. (2012)	Predictive modeling of wheat pest impact	Regression models (RF, RKHS, Bayesian LASSO, MLR, Ridge)	Compared predictive accuracy for pest-related outcomes	Dataset-specific results; limited field deployment and interpretability

However, traditional machine learning techniques, though showing good results when processing agricultural images, suffer from significant drawbacks. Being based on manually engineered features, they do not possess the capability of automatic feature extraction. Thus, traditional ML techniques fail to capture subtle nuances and peculiarities of the data when performing image classification.

### 2.2 Deep learning-based methods

DL algorithms like CNN have experienced rapid advancements and have proved effective for image classification. By allowing multi-level feature learning of input data, DL eliminates limitations inherent in classical ML algorithms with regards to handcrafted features. In terms of agriculture image analysis, DL methods have been demonstrated as highly accurate in capturing necessary features for advanced computer vision applications. Transfer learning refers to an adaptation of a model pre-trained on one problem to solving another problem. It exploits a model’s knowledge of source domain data, eliminating the need for training from scratch. It is highly recommended when there is not enough data for target task training. It leverages the knowledge obtained from large data source domains.

According to Padshetty et al., the Leaky Rectilinear Residual Network is a modified version of ResNet architecture incorporating the Leaky ReLU activation function to address issues of vanishing gradients and dead neurons in networks. This network exhibited good performance in detecting different diseases in plants. However, it still remains too computationally expensive for low-resource environments. Another study by Bao et al. presents a lightweight CNN inspired by MobileNet and GhostNet networks with focus on reducing the model parameter number while maintaining high accuracy of prediction. Inferences from their model were much faster, but this reduction in model capacity may pose problems when working with highly complicated or large-scale dataset of diseases.

As for Amin et al., they proposed EfficientNet-B0 and DenseNet-121 models and feature concatenation technique for extracting features from diseased corn leaf images. By comparing the performance of models, they found that feature fusion resulted in increased accuracy of prediction. However, it greatly increased the computational complexity of the process. In their study, Anwar et al. used ensemble learning for developing a CNN model capable of classifying agricultural pests. They used four pre-trained CNN models, namely ResNet, VGG, Xception, and Inception, and created ensembles of different models to improve classification accuracy. The ensemble of VGG16, VGG19, and ResNet50 achieved best accuracy among the single models, proving the power of this strategy. Yet, an ensemble of CNN models increases inference time and model size.

Furthermore, Timothy et al. applied the ResNet50-based CNN for the detection of cashew leaf, nut, and stem diseases. By training the model on images acquired from the LAUTECH Teaching and Research Farm, they showed high average accuracy (97.76%). Nevertheless, the dataset used in the experiment had rather a narrow geographical distribution that could affect the generalizability of the classifier. Similarly, Vidhya and Priya tested the efficiency of the state-of-the-art real-time object detection algorithm (YOLOv5) in identifying Tea Mosquito Bug (TMB) infestations in cashew plants. The authors also developed and provided a novel high-resolution dataset (640 × 640 pixel images) that included images taken at Kerala Agricultural University, Thrissur. The model successfully identified the presence of TMBs; however, the success of the detection heavily depends on the conditions in which images are taken.

Further enhancement of their model was done by Palaniappan et al. via application of the Enhanced Linear Discriminant Analysis (ELDA) – a median-based algorithm that minimizes the influence of outliers on class means for classification purposes. By applying the proposed classifier, they achieved 97.7% accuracy on CCDDB, 99.8% on PDDB, and 81.7% on a mixture of pests and diseases. Therefore, the ELDA model performs well consistently, yet performance drops considerably for heterogeneous datasets. On the contrary, Panchbhai et al. improved their earlier research in terms of reducing model complexity by modifying MobileNetV2. Namely, they used average pooling in the last layer of the network, thus achieving almost perfect accuracy (99.8%) and having a smaller parameter size. However, their model was only validated in a lab environment.

Finally, Hamza et al. introduced a Bayesian-optimized Deep CNN with the interpretability element. For this purpose, they adopted EfficientNet-B0 for feature extraction in order to apply a slice-based sequence fusion method for classification. They also used the Grad-CAM technique for the visualization of key regions of interest. Although the algorithm exhibited excellent performance, it was specifically designed for medical imaging purposes and should be adjusted for use in an agricultural context.

The classification of crop diseases and pests is limited by several factors, which hinder development of highly accurate classification systems:

- Most researchers utilize classical CNN architectures that are capable of extracting good features from image data but have lots of parameters and cannot capture long-range dependencies observed in crops and pests;
- The majority of works apply conventional CNNs or employ transfer learning without adapting them to the particular nature of agricultural datasets. Thus, models’ adaptability to new data is limited by this issue;
- Lastly, most works are based on single-path CNN models that can result in false positive/negatives.

Thus, the solution to these problems will help develop a better system that would be more accurate and robust when predicting the presence of diseases or pests. The goal of this work is to create an algorithm that allows improvement in predictive accuracy through the combination of two deep learning architectures and fusion of their features. In contrast to (Geetha et al., 2020) (Padshetty and Ambika, 2023), and (Bao et al., 2022), the proposed solution will integrate features of multiple CNN architectures in order to enhance the model’s performance. Feature fusion helps to obtain more informative and representative features of crop disease images, thus minimizing possible biases. **Table 2** outlines unique features of the discussed papers focusing on the cashew crop.

**Table 2:** Summary of individual articles on deep learning based pest detection and classification.

Ref.	Study focus	Methodology/model	Outcome/results	Drawbacks/limitations
Padshetty et al (Padshetty and Ambika, 2023)	Plant leaf disease detection	Leaky Rectilinear Residual Network (ResNet + Leaky ReLU)	High classification accuracy; improved gradient stability	Computationally intensive; not optimized for mobile/edge devices
Bao et al. (2022)	Lightweight plant disease model	Compact CNN inspired by MobileNet & GhostNet	Reduced parameters with competitive accuracy	Limited representational capacity on complex datasets
Amin et al. (2022)	Corn leaf disease detection	Feature concatenation using EfficientNet-B0 & DenseNet-121	Improved feature richness and accuracy	Increased computational cost and memory use
Anwar et al (Anwar and Masood, 2023)	Crop pest classification	Ensemble of ResNet, VGG, Xception, and Inception	Ensemble (VGG16+VGG19+ResNet50) achieved best accuracy	Large model size and slow inference speed
Timothy et al (Mathew et al., 2022)	Cashew disease classification	CNN based on pre-trained ResNet50	97.76% accuracy on cashew leaf, nut, and stem images	Dataset limited to one region; possible overfitting
Vidhya & Priya (Vidhya and Priya, 2024)	Detection of Tea Mosquito Bug in cashew	YOLOv5 with custom 640×640 dataset	Effective early detection of TMB infestations	Dependent on high-quality, controlled imaging
Sudha & Kumaran (Palaniappan et al., 2024)	Cashew leaf disease classification	Enhanced Linear Discriminant Analysis (ELDA)	97.7% (CCDDB), 99.8% (PDDB), 81.7% (mixed dataset)	Sensitive to heterogeneous datasets and outliers
Panchbhai et al. (2024)	Cashew fruit and nut disease recognition	Modified MobileNetV2 (CASMODMOBNET)	99.8% average accuracy with reduced parameters	Limited validation; tested in controlled environments
Hamza et al. (2022)	COVID-19 classification (interpretable AI)	Bayesian-optimized DCNN + EfficientNet-B0 + Grad-CAM	High interpretability and robust classification	Domain-specific; limited transferability to plant disease tasks

## 3 Proposed work

**Figure 5:**
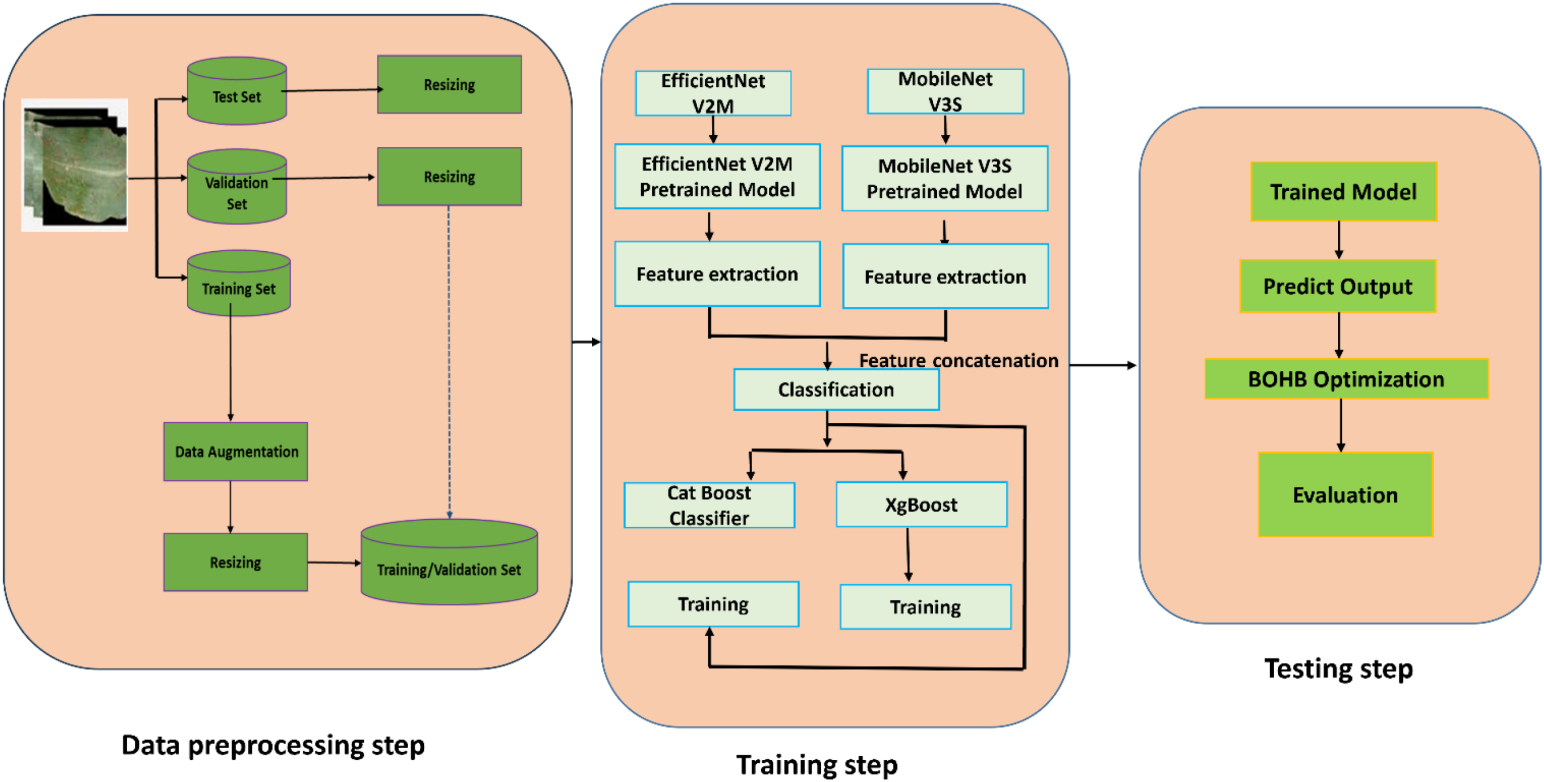
Proposed hybrid fused classification model.

The process of cashew growing is also very sensitive to different kinds of diseases that affect the leaves, leading to yield losses. Although deep learning techniques have shown promise in plant disease identification, many existing studies rely heavily on conventional CNN architectures, which, despite their strong feature extraction capabilities, involve many parameters and fail to effectively capture long-range dependencies present in crop and pest images. Many deep learning work involved feature concatenation which resulted in enhanced accuracy but also increased computational complexity, and ensemble frameworks may provide superior accuracy but suffer from longer inference times and larger model sizes. Variations in illumination, background, and disease symptoms further hinder model robustness. In addition, a significant portion of current work depends on single-path architectures, which often lead to suboptimal performance, particularly with respect to high false negative and false positive rates To address these challenges, this study proposes a computationally efficient hybrid late-fusion framework that integrates complementary deep learning backbones with optimized boosting classifiers to achieve improved accuracy and adaptability for cashew leaf disease recognition. **Figure 5** shows the overall framework of this model.

To this end, we propose a hybrid late-fusion architecture that integrates two complementary convolutional backbones—EfficientNetV2-M and MobileNetV3-S—for feature extraction, followed by two classifiers (1) XGBoost and (2) Catboost. The rationale behind utilizing two architectures—(1) EfficientNetV2-M + MobileNetV3-S with XGBoost classifier and (2) EfficientNetV2-M + MobileNetV3-S with CatBoost classifier—for cashew leaf disease detection lies in the complementary strengths of each combination, enhancing overall classification performance. The first architecture is based on the use of EfficientNetV2-M due to its effective scaling techniques that help to extract fine-grained features, together with MobileNetV3-S, which ensures fast computations for real-time applications. With the help of the XGBoost classifier, such an ensemble model benefits from XGBoost’s ability to work efficiently with high-dimensional data and resist overfitting while being able to construct interpretable models. The second architecture is based on the similar combination of EfficientNetV2-M and MobileNetV3-S for feature extraction but uses CatBoost as the classifier. The classifier shows excellent results when dealing with categorical features with little or no preprocessing required and demonstrates high robustness to the problem of overfitting and imbalanced dataset that is usually a challenge in agrarian contexts.

**Figure 6:**
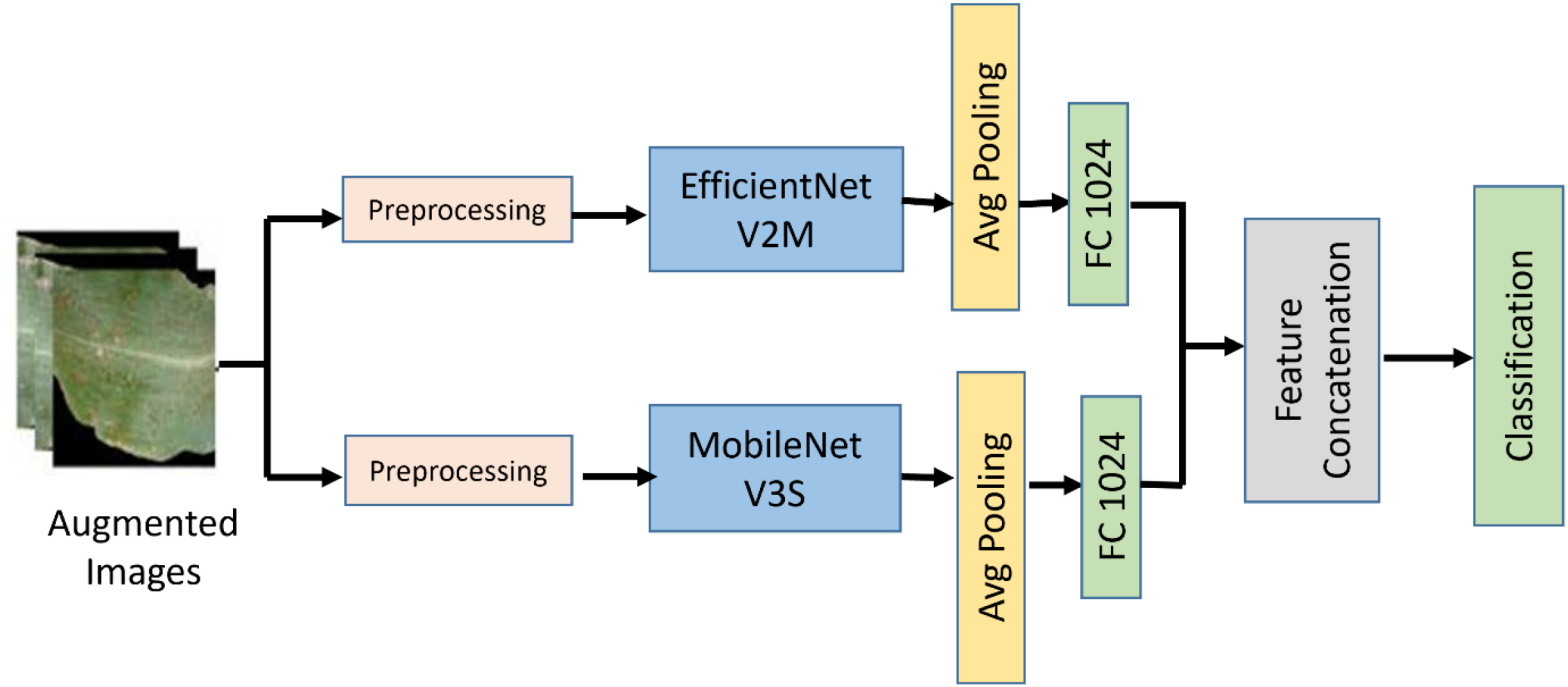
Classification model.

Such architectural diversity helps to tackle different issues of disease classification in order to make the architectures appropriate for practical application in precision agriculture and ensure reliable diagnosis of cashew leaf disease. Using two state-of-the-art architectures for building the ensemble model helps to extract a wide variety of distinctive features that are necessary for identifying the most complex patterns of cashew leaf diseases. The whole model is optimized using the method called Bayesian Optimization with HyperBand (BOHB) to obtain optimal model performance and generalizability. The architecture of the proposed methodology is illustrated in **Figure 6**.

### 3.1 Methodology

#### 3.1.1 Hybrid late-fusion architecture

The proposed hybrid architecture is organized as a two-stage pipeline: (i) dual-backbone feature extraction and (ii) late fusion followed by boosting-based classification (**Figure 6**). The following subsections describe each component in detail and justify the design choices.

#### 3.1.2 Dual-backbone feature extraction

Two complementary CNN backbones are used simultaneously to capture both fine-grained features and compact, computationally efficient representations. Transfer learning was employed for both EfficientNetV2-M and MobileNetV3-S backbones. During training, the initial layers of each network were frozen to retain generic feature representations learned from large-scale datasets. Only the final blocks of the networks were unfrozen for fine-tuning. Specifically, the last 2 blocks (24 MB conv blocks) of EfficientNetV2-M and the last 3 bottleneck layers (9 Bottleneck (bneck) blocks) of MobileNetV3-S were set as trainable, while the remaining layers were kept frozen.

A differential learning rate strategy was adopted to stabilize training. The fine-tuned backbone layers were trained with a lower learning rate of 1 × 10⁻⁵, while the newly added projection, fusion, and fully connected layers were trained with a higher learning rate of 1 × 10^-4^. This technique helps in adapting the pretrained features gradually and supports rapid learning in task-specific layers. Selective fine-tuning prevents overfitting, improves convergence stability, and guarantees effective exploitation of pretrained features for classifying diseases on cashew leaves.

#### 3.1.3 EfficientNetV2M

EfficientNetV2-M is selected due to its ability to scale up the architecture using three factors – depth, width, and resolution – together to create a compound scaling model. This model is effective at extracting texture, lesion boundary, and color gradient features that typically distinguish cashew diseases. EfficientNetV2-M uses the concept of transfer learning. Pretrained model weights are used for initialization, while the last layer(s) undergoes fine-tuning using the CCMT data for adjusting low and middle-level filters specific to cashews.

#### 3.1.4 MobileNetV3-S (lightweight/global features)

MobileNetV3-S provides a resource-efficient feature extractor that captures broader shape, color distribution, and coarse structural cues. Its depthwise separable convolutions and squeeze-and-excitation (SE) modules keep FLOPs and parameter counts low, making the pipeline suitable for deployment on constrained hardware if needed.

MobileNetV3-S provides an efficient feature extractor that can capture global shape information, color distribution, and coarse structures. Its depthwise convolution operations and squeeze-and-excitation blocks ensure minimal floating point operations (FLOPs) and parameters, making the network efficient enough to run on limited computational resources.

The backbone networks (EfficientNetV2-M and MobileNetV3-S) process the input image independently, and the Global Average Pooling (GAP) step is applied to generate a compact feature vector. Then, a fully-connected layer with 1024 neurons is used to project features extracted from both backbone networks to ensure normalization and alignment of backbone-specific features. Next, the two feature vectors are concatenated to create a combined feature vector. To further minimize the dimension of the combined vector and maximize its discriminative power, the fused feature vector is projected into a 512-dimensional vector. The generated feature vector will then be fed into the classifiers (XGBoost and CatBoost). This is depicted in **Figure 6**.

Each of the backbones receives the identical input image $x\in {\mathbb{R}}^{H\times W\times 3}$ (where H = W = 224 in the experiment) and produces a tensor of high-level features. For the backbone i∈1,2, the output tensor is represented as follows in Equation 1.

(1) $$T_{i}\in {\mathbb{R}}^{h_{i}\times w_{i}\times c_{i}}$$

where $h_{i},w_{i}\text{ and }c_{i}$ are the spatial dimensions and channel depth at the selected stage (commonly the output of the last convolutional block).

For obtaining feature vectors that are compact, a global aggregation method is applied:

Apply global average pooling (GAP) to T*_i_* to produce a vector $v_{i}\in {\mathbb{R}}^{c_{i}}$: It is determined by using Equation 2.

(2) $$v_{i}=\text{GAP}\left(T_{i}\right)=\frac{1}{h_{i}w_{i}}\sum_{u=1}^{h_{i}}\sum_{v=1}^{w_{i}}T_{i}\left(u,v\right)$$

Further, we applied a small fully connected (FC) projection with Batch Norm and nonlinearity to normalize and reduce dimensionality as represented in Equation 3.

(3) $${\tilde{v}}_{i}=\sigma \left(\text{BN}\left(W_{i}v_{i}+b_{i}\right)\right),{\tilde{v}}_{i}\in {\mathbb{R}}^{d}$$

d is defined as the selected embedding dimension size; for example, we have set d = 512 in our experiments. In addition, σ(·) refers to the ReLU activation function, while BN refers to batch normalization. Through this mapping technique, the outputs of the two networks become the same dimensional size d, making the fusion process easier and preventing any dominance by either network because of their different dimensional sizes.

#### 3.1.5 Feature alignment and pre-fusion processing

Before performing fusion, the vector representations ${\tilde{v}}_{1}\text{ and }{\tilde{v}}_{2}$ are normalized and regularized separately to make training more stable. Specifically, the L2 normalization is used for deriving the discriminating feature as illustrated by Equation 4.

(4) $${\hat{v}}_{i}=\frac{{\tilde{v}}_{i}}{∥{\tilde{v}}_{i}{∥}_{2}+\epsilon }$$

These steps encourage balanced contribution from each backbone and mitigate overfitting, particularly when dataset size is moderate.

#### 3.1.6 Late fusion mechanism

We adopt concatenation as the primary late-fusion mechanism, producing a unified feature vector:

(5) $$F=\left[{\hat{v}}_{1}∥{\hat{v}}_{2}\right]\in {\mathbb{R}}^{2d}.$$

The method of concatenation retains all the information contained within the two embeddings, while also offering interpretability. Equation 5 illustrates the concatenation mechanism. In order to improve discriminative capabilities and eliminate any redundancy after concatenation, a fusion projection layer is used. This layer is represented in Equation 6.

(6) $$z=\sigma \left(\text{BN}\left(W_{f}F+b_{f}\right)\right)\in {\mathbb{R}}^{d_{f}},$$

where *d_f_* is the fusion embedding size (e.g., *d_f_* = 512). The purpose of the fusion projection layer includes: (i) reducing the dimensions of the fused embedding, (ii) weighting the contributions of different backbones in the process, and (iii) creating a small feature vector that can be used in other classifiers. The final fused feature vector F is independently passed as input to two models, namely XGBoost and CatBoost, both of which are capable of capturing nonlinear relationships between features. Training and evaluating each model separately allows for the comparison of the results achieved by XGBoost and CatBoost and choosing the best one for the presented framework.

### 3.2 BOHB optimization

The BOHB algorithm is used for optimizing both classifier hyperparameters and the backbone parameters (EfficientNetV2M and MobileNetV3S). To increase efficiency in the process of parameter tuning, the study proposes combining the global search capabilities of Bayesian Optimization with the efficient resource usage of Successive Halving in HyperBand. The main goal of BOHB optimization is to obtain the best possible classification accuracy on the validation subset of CCMT Cashew Leaf Disease Dataset by minimizing the optimization time and overfitting. To assess each configuration, the validation accuracy based on 5-fold cross-validation is calculated. The optimization process is conducted by repeating the same sequence of steps: 1) Initialization, 2) Low-budget evaluation, 3) Successive halving, 4) Bayesian update, and 5) Refinement. First, the initial set of hyperparameter configurations is generated using random sampling in the specified search space. All configurations are trained on a low computational budget corresponding to only one ninth of the total maximum budget B_max, and their validation accuracies are computed. Next, one-third (η⁻¹) of the top-performing candidates are retained and retrained using a higher budget of computation resources. The surrogate model is then updated using all collected data to form the basis for generating new hyperparameter sets for training. Finally, the above steps are performed again and again until the maximum budget is reached, or there is no improvement in consecutive iterations. Such an approach allows directing the computational power towards the most promising configurations of hyperparameters, thus providing the right balance between exploring (sampling from the search space) and exploiting (optimizing the promising candidates) the latter.

**Algorithm 1** illustrates the process of BOHB optimization used to tune the hybrid model. Initially, a surrogate model M predicts promising areas in the parameter space. Using Successive Halving, each configuration is ranked based on its performance and the best-performing third is retained for further evaluation on an increasing budget. The procedure is repeated until the maximum budget *B_max_* is reached and the resulting optimal set the optimal set θ* is selected.

Algorithm 1 **BOHB optimization workflow.**

Input:θ ∈ Θ → Hyperparameter search spaceη → Downsampling factor (default = 3)Bmin, Bmax → Minimum and maximum training budgetsN → Number of configurations per iterationOutput:θ* → Optimized hyperparameter configuration1: Initialize surrogate model M (Tree-structured Parzen Estimator)2: Initialize observation set D ← ∅3: for each iteration t = 1 to T do4: Sample N configurations {θ_1_, θ_2_, …, θ_N} from Θ using M5: Assign initial budget b ← Bmin6: while b ≤ Bmax do7:   Train each configuration θ_i for budget b8:   Evaluate validation accuracy Acc_i(θ_i)9:   Rank configurations by Acc_i(θ_i)10:   Retain top ⌈N/η⌉ configurations11:   Update D ← D ∪ {(θ_i, Acc_i(θ_i), b)}12:   Update surrogate model M using D13:   Increase budget: b ← η × b14: end while15: end for16: Select θ* = argmax_{θ_i ∈ D} Acc_i(θ_i)17: return θ*

By employing BOHB optimization technique, the proposed approach was able to optimize the hybrid model’s parameters along with the boosting layers for achieving quick convergence. Experimental evaluation revealed that BOHB-optimized parameter settings performed significantly better than those set manually as well as through grid search methods, providing an improvement of about 2%-3% in the validation accuracy and less overfitting on new test samples. This is made possible by employing BOHB optimization to ensure that the hybrid model remains accurate, efficient, and resilient even when faced with different data situations.

BOHB optimization of hyperparameter Θ refers to the optimization of both the parameters of the boosting algorithm as well as those used to tune the feature extractors of the model. **Table 3** below shows the major parameters optimized by BOHB.

**Table 3:** Hyperparameters values.

Component	Hyperparameter	Search range
XGBoost	Learning rate	[0.001 – 0.3]
	Maximum depth	[3 – 10]
	Number of estimators	[100 – 800]
	Subsample ratio	[0.5 – 1.0]
	Regularization (λ, α)	[0 – 10]
CatBoost	Learning rate	[0.001 – 0.3]
	Maximum depth	[3 – 10]
	L2 regularization	[0 – 5]

## 4 Results and analysis

### 4.1 Experimental setup

The structure of the proposed model was designed with the aid of the Keras library. Using deep learning models becomes simple with Keras, which is a Python library for creating neural networks. This library makes the process of designing, training, and testing the models much easier. The creation, training, and testing of the models were carried out with the help of Google Colaboratory (Colab). It has the GPU along with two CPU cores and 12 GB of RAM.

### 4.2 Dataset for the study

Cashew, Cassava, Maize and Tomato (CCMT) (https://www.kaggle.com/datasets/siddharthjiyani/ccmt-dataset-for-crop-pest-and-disease-detection/data) crop pest and disease detection was carried out using raw (24,881 images) and augmented (102,976 images) color images covering twenty-two (22) classes. The images included in the dataset were taken from natural environments, hence causing differences in factors such as lighting, background, leaf orientation, and the nature of the disease manifestation itself. These variations will pose some level of difficulty for the model to generalize well; however, this will make the data set closer to reality. The research considers only cashew plant images sized 400x400, which are considered in this study.

The cashew data set is made up of four diseases, including Anthracnose, Gummosis, Leaf Miner, and Red Rust, apart from the disease-free plant samples (Kumari et al., 2019). Anthracnose, a fungal infection that comes about through the pathogen Colletotrichum gloeosporioides, occurs in dark brown and sunken leaf and fruit spots, hence causing yield losses in the humid areas where the crop grows. The disease of Gummosis, whose causative organism is mainly Lasiodiplodia theobromae, is characterized by a gum discharge from the bark of the trees; in extreme cases, this leads to death of branches and the whole tree. Leaf miner attacks, which involve the larvae of moths like Acrocercops syngramma, occur when tunnels form inside leaf tissues. Red Rust, an algal infection caused by Cephaleuros virescens, is recognized by reddish-orange spots on leaves and stems, gradually weakening the plant’s vigor. The dataset additionally has images of healthy samples, facilitating the classification of infected images from others. The augmentation methods of rotating, shearing, zooming, and horizontal flipping were only performed on the training dataset after segmentation. The validation dataset was extracted from the training dataset before any form of augmentation. This is important in ensuring that there is no duplication of any images within the three datasets, hence avoiding data leakage. The corresponding values are displayed in **Table 4** for every augmentation method that was utilized. In the last stages, the images were resized to 400 by 400 pixels before the subsets were utilized.

**Table 4:** Dataset -size.

S. no	Pest disease	Images	
		Raw data	Augmented data
1	Anthracnose	1701	4,940
2	Gummosis	392	2,139
3	Healthy	1368	7,213
4	Leaf miner	1358	4,953
5	Red rust	1682	6,566

**Figure 7:**
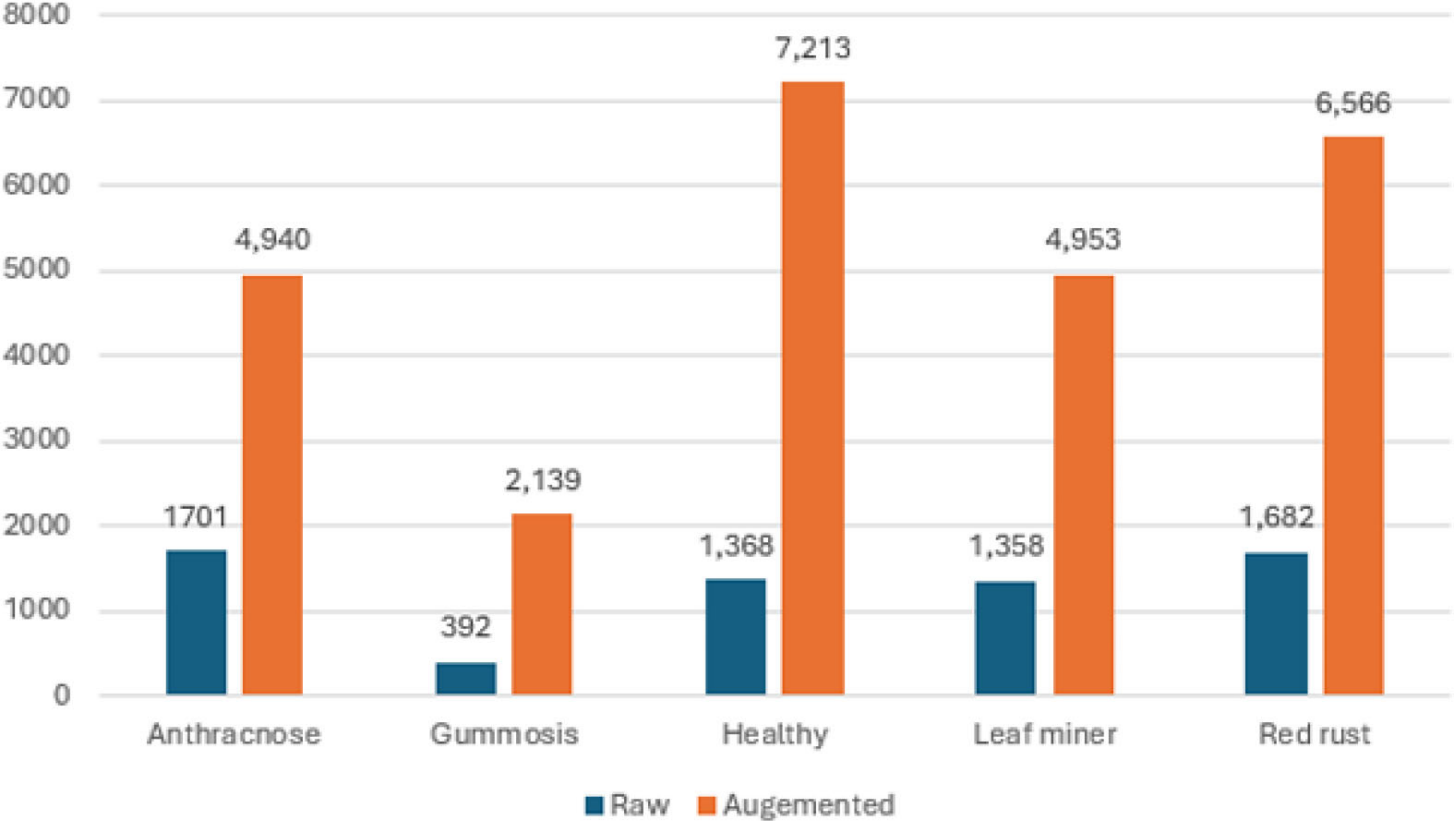
Raw and augmented dataset population.

**Figure 8:**
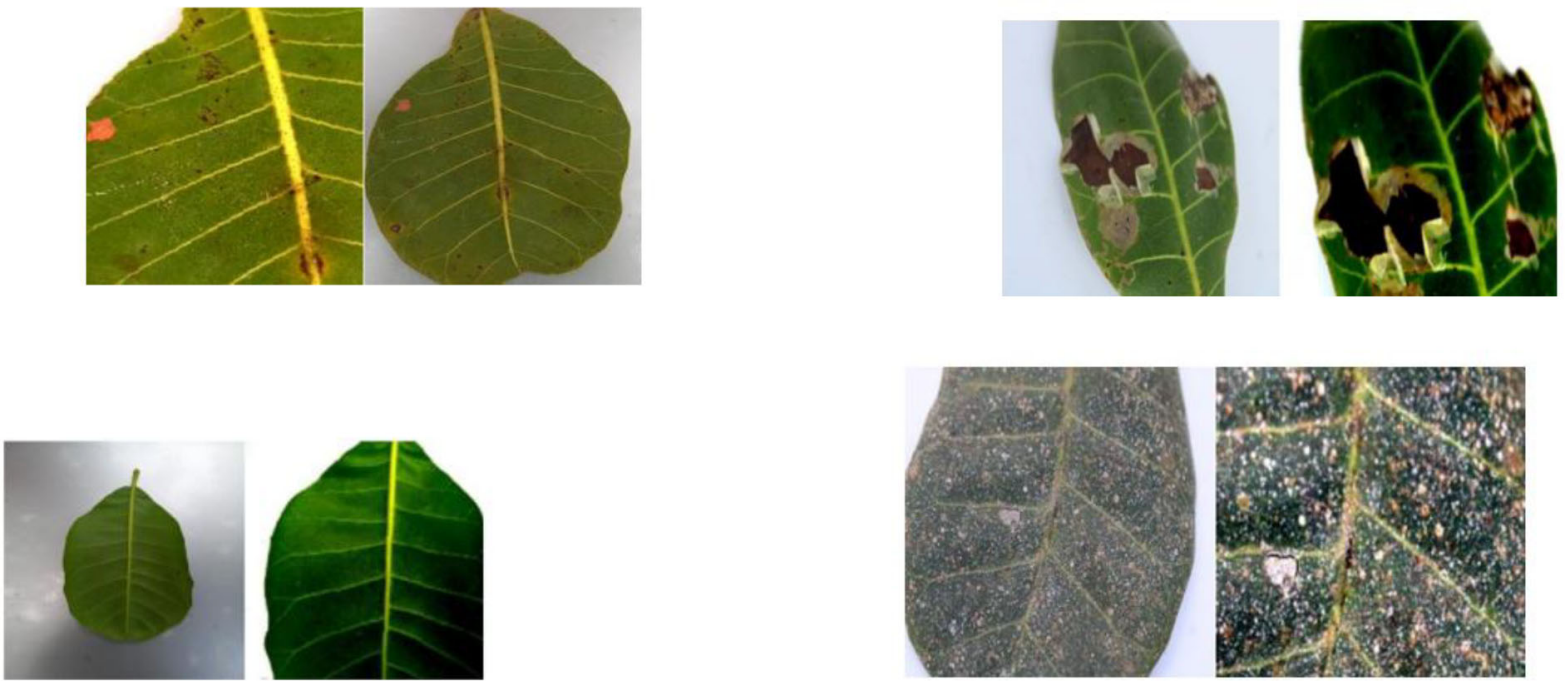
Original image vs. augmented images.

The population distribution of the raw and augmented images of the different classes in the dataset is shown in **Figure 7**. The original image and augmented images are shown in **Figure 8**. The proposed classification model and its several stages are described in this section. The proposed structure is visually represented in **Figure 5**, which is divided into three primary stages: (1) Data preprocessing, (2) Training step, and (3) testing step.

**Figure 9:**
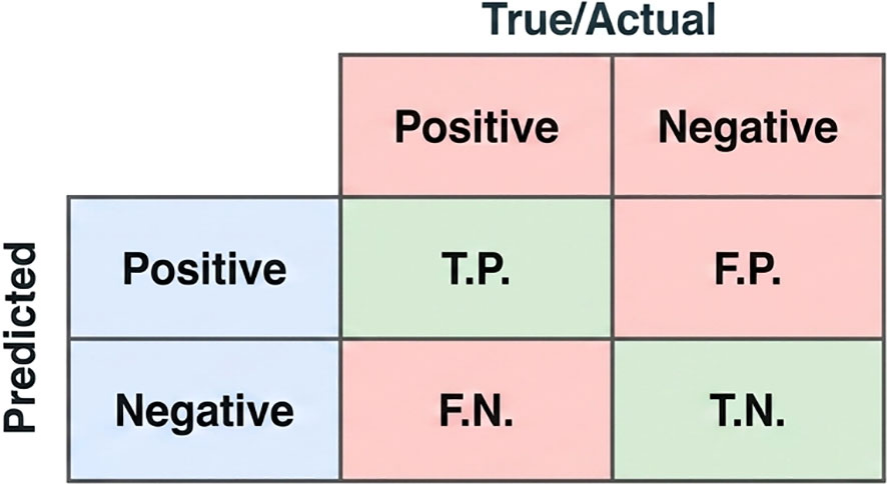
Confusion matrix. A prediction’s outcome might be categorized as TP, TN, FP, FN.

### 4.3 Feature extraction and training phase

The dataset was partitioned into an 80:20 ratio, where 80% of the samples were allocated for training and 20% for testing. From the training set, 20% of the samples were further separated to form a validation set. To avoid data leakage and ensure unbiased evaluation, the dataset was initially split into training (80%) and testing (20%) subsets using only the original images. The augmentation methods of rotating, shearing, zooming, and horizontal flipping were only performed on the training dataset after segmentation. The validation dataset was extracted from the training dataset before any form of augmentation. This is important in ensuring that there is no duplication of any images within the three datasets, hence avoiding data leakage. The training subset allowed the model to extract complicated and discriminative image features. In contrast, the validation subset, kept independent of training, was employed to monitor model performance after each epoch and to guide optimization by preventing overfitting. Finally, the test subset was utilized only after the completion of training, providing an unbiased evaluation of the model’s generalization ability on previously unseen data. **Figure 5** illustrates the fused CNN’s architecture, which was utilized in the experiment. The new proposed model was built using the MobileNetV3S and EfficientNetV2 as baseline model. After loading each model’s pretrained weights, the models’ classification halves were replaced with an average pooling layer and a fully connected layer of 1024 neurons. Further the classification is performed by XGBoost and CatBoost Classification models. Both models are tuned with Bayesian Optimization and Hyperband (BOHB) method.

Xtreme Gradient Boosting (XGBoost) is a powerful ensemble learning algorithm based on gradient-boosted decision trees, widely used for classification tasks due to its scalability, speed, and ability to handle complex data patterns. It works by iteratively training weak learners (decision trees) and combining them to form a strong predictive model that minimizes classification errors. In this study, features are first extracted using fused hybrid model, and the resulting feature vectors are transformed into NumPy arrays to serve as inputs for the XGBoost classifier.

CatBoost is a gradient boosting algorithm developed by Yandex, designed to handle both numerical and categorical features efficiently. In contrast to conventional boosting algorithms, CatBoost implements advanced methodologies such as ordered boosting and categorical variable encoding to minimize prediction error and avoid overfitting. The native capability of handling categorical features with minimal preprocessing ensures that CatBoost excels in classification problems. In the proposed research, feature vectors derived from the fused hybrid model are transformed into NumPy arrays and fed into the CatBoost classifier.

Bayesian Optimization and Hyperband (BOHB) is a novel combination of hyperparameter optimization approaches. On one hand, BOHB is built on Bayesian optimization, an intelligent optimization algorithm that intelligently selects optimal hyperparameters. On the other hand, Hyperband, which allows the process to terminate underperforming configurations early, significantly reduces the computational effort. Consequently, BOHB is highly efficient as it rapidly converges toward optimal hyperparameters. In this research, BOHB is employed to tune the hyperparameters of the classifier, thus increasing efficiency and accuracy compared to other conventional optimization algorithms such as grid and random searches.

### 4.4 Implementation details

The following configurations were used for the training process of the CNN backbone models:

- Optimizer: Adam
- Initial learning rate: 0.0001
- Batch size: 32
- Epochs: 50 (with early stopping patience = 10)

First, transfer learning was applied, where the convolutional base of EfficientNetV2-M and MobileNetV3-S was pre-trained using the weights of pre-trained models. Next, the top layers of the model, consisting of the classification layers, were substituted by global average pooling followed by a dense layer containing 1024 neurons. For the boosting classifiers the hyper parameters are specified in **Table 3**.

XGBoost: learning rate (0.15), max depth (4), number of estimators (250), subsample (0.36).

CatBoost: learning rate (0.1–0.3), depth (3–10), L2 regularization (0–5).

Hyperparameters were optimized using the BOHB method with 50 trials and 5-fold cross-validation on the training set. These settings ensure a balance between model performance and computational efficiency.

### 4.5 Evaluation phase

The model’s performance was assessed using the test set to ensure that it didn’t overfit during the evaluation stage. To evaluate the proposed approach, 20% of the original dataset sample is used as the test set. Correctly classifying an input image as belonging to the northern leaf blight, common rust, gray leaf spot, or healthy classes served as the basis for the evaluation.

## 5 Results and discussions

This section provides a detailed discussion of the proposed research’s findings. For evaluating the proposed hybrid model, the following evaluation metrics are used which are outlined.

### 5.1 Evaluation metrics

The performance of the trained models was evaluated using metrics which are accuracy, precision, recall, and F1-score, along with the Receiver Operating Characteristic (ROC) curve and the Area Under the Curve (AUC). These measures together provide a detailed understanding of the model’s classification ability. **Figure 9** shows the confusion matrix which is used to calculate this metrics.

Accuracy: A model’s overall accuracy in this context is determined by dividing the number of successfully predicted samples by the total number of predictions (Equation 7).

(7) $$accuracy=\frac{TP}{TP+TN+FP+FN}$$

Precision: The ratio of correctly predicted positives to all positive predictions for a class is known as precision. (Equation 8).

(8) $$precision=\frac{TP}{TP+FP}$$

Recall: The fraction of all positive samples that are accurately predicted to be positive is measured by recall, which is determined by Equation 9.

(9) $$recall=\frac{TP}{TP+FN}$$

Additionally, the weighted average of Precision and Recall is used to calculate the f1-score, as shown in Equation 10.

(10) $$f1-score=\frac{2*precision*recall}{precision+recall}$$

**Figure 10:**
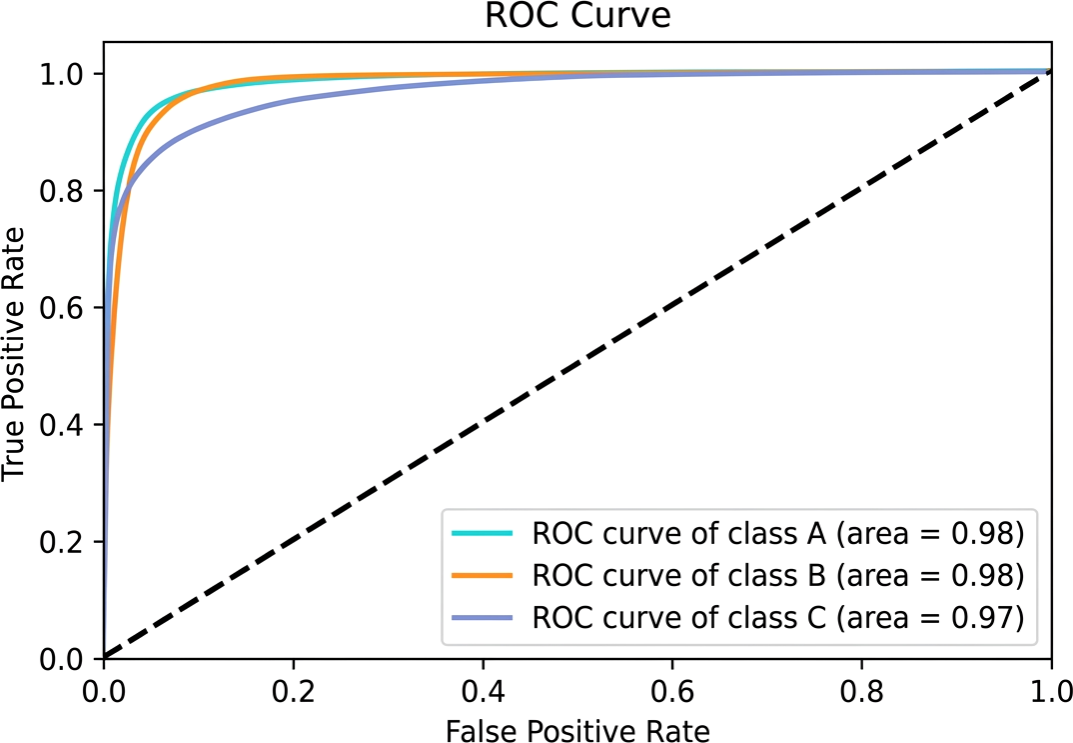
The receiver operating characteristic curve for a multi-class classification.

Received Operation Characteristics (ROC-AUC): The Receiver Operating Characteristic–Area Under the Curve (ROC-AUC) measures how well a classification model can differentiate across classes, by measuring the area under the curve formed by plotting the true positive rate against the false positive rate across varying decision thresholds. Interpretation of ROC-AUC: AUC = 1.0 → Perfect classification. AUC = 0.5 → No discriminative ability (random guessing). 0.5< AUC< 1.0 → Increasingly better class separability. A one-vs-all method is used to construct the ROC curve for each class in multi-class classification, using the chosen class as the positive class and all others as the negative class. **Figure 10** shows the ROC curve for multi class classification.

### 5.2 Model evaluation

After augmenting the original training subset, the CNN represented in **Figure 8** was created and trained with the training set. The learning rate at the start of the training was 0.0001. The validation loss value was programmed to automatically drop by 0.1 every four epochs for a total of 50 epochs in which there was no improvement. For preventing overfitting, the early stopping technique was applied after the models didn’t improve for ten epochs.

**Figure 11:**
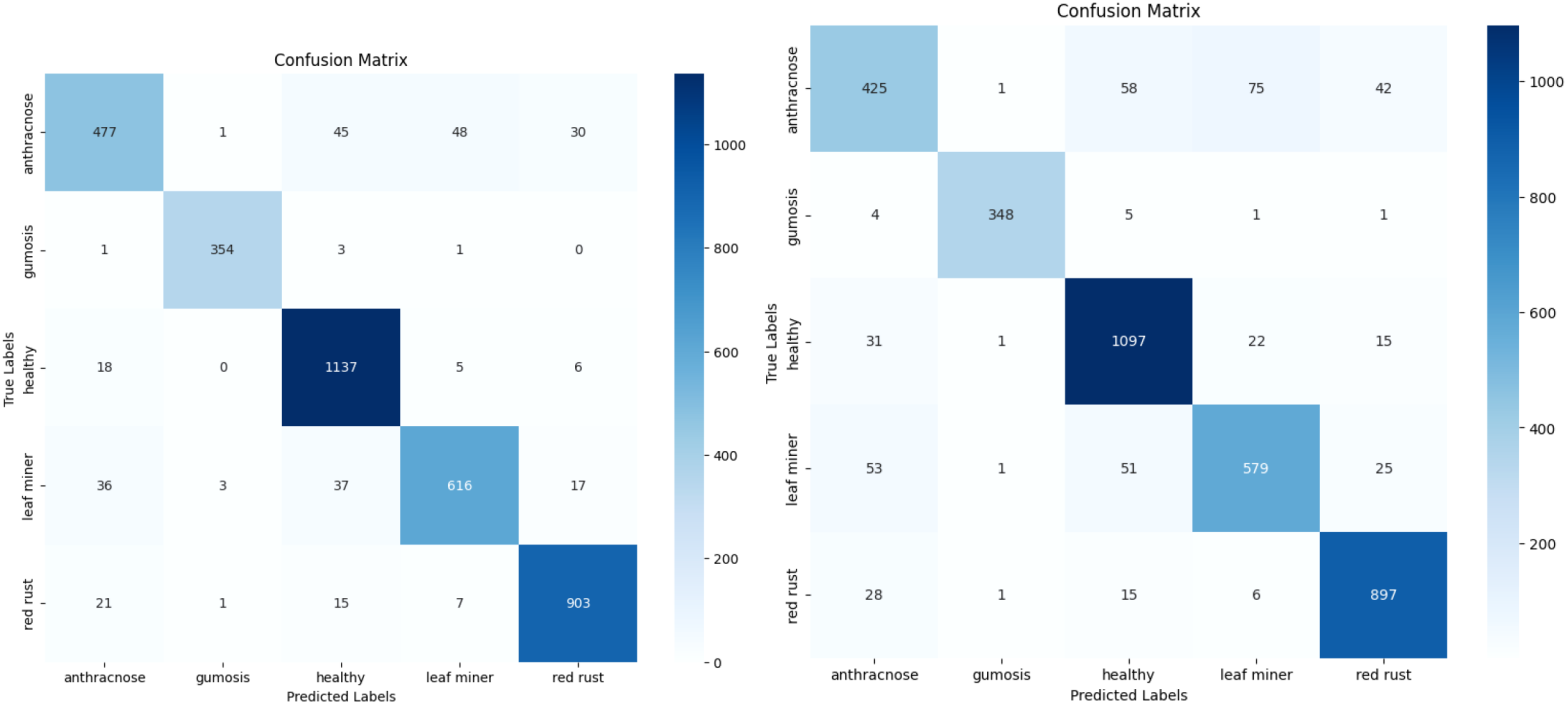
Confusion matrices – XGBoost and CatBoost classification.

**Figure 12:**
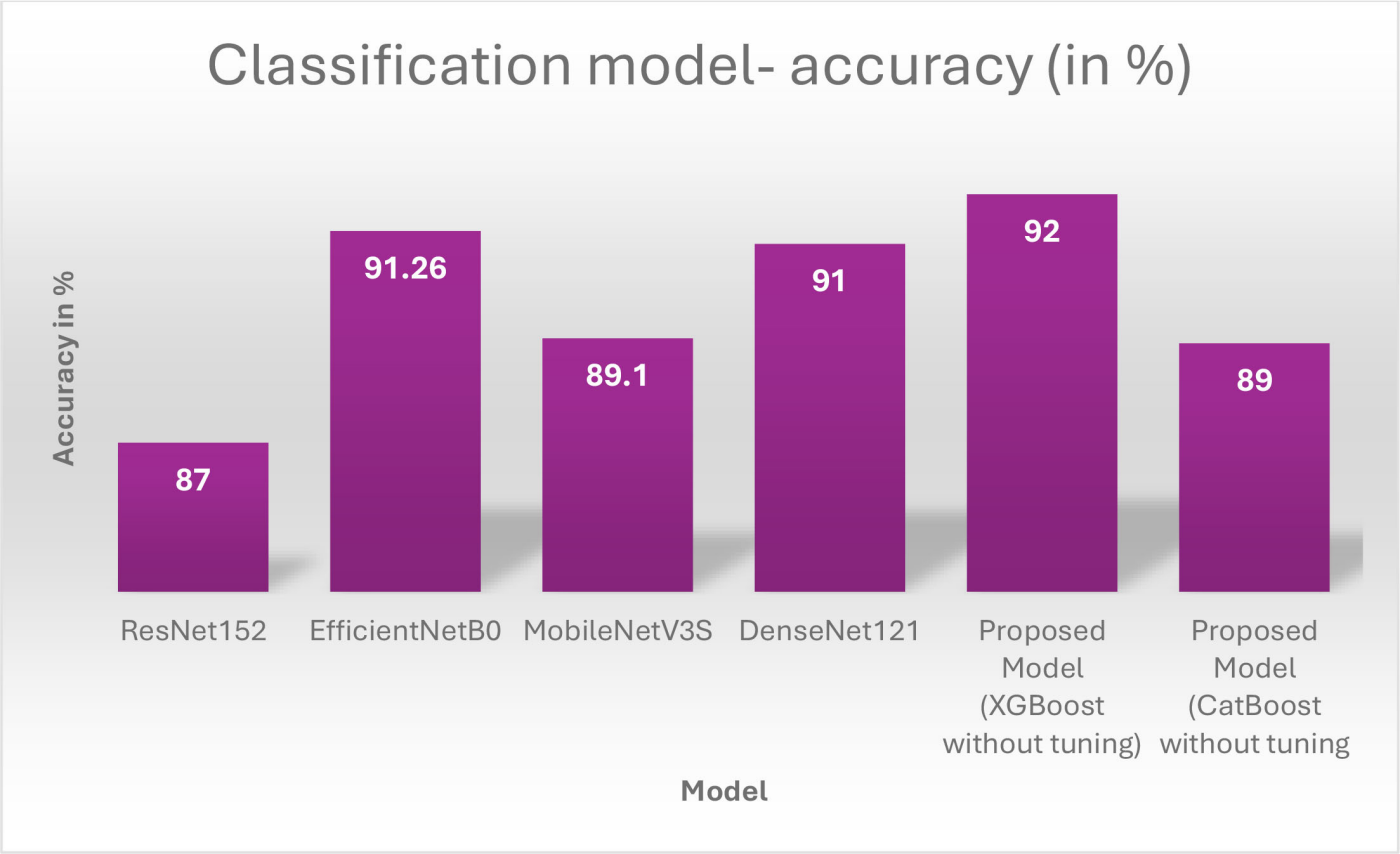
Performance comparison – before optimisation.

Once training is complete, the models are tested against the test subset to assess their performance. On the test set, the proposed model achieved a classification accuracy of 93.10%. The confusion Matrix of both XGBoost and CatBoost classification models are depicted in **Figure 11**. A comparison of accuracy, training time and testing time is presented in **Table 5** and illustrated in **Figure 12**, demonstrating that the proposed model achieving the highest accuracy.

**Table 5:** Performance comparison – before optimization.

S. no	Model	Accuracy (in %)	Training time (in sec)	Testing time (in sec)
1	ResNet152	87	15.8	0.62
2	EfficientNetB0	91.26	21.2	0.45
3	MobileNetV3S	89.1	11	0.4
4	DenseNet121	91	12	0.8
5	Proposed Model (XGBoost without tuning)	92	8.04	0.18
6	Proposed Model (CatBoost without tuning	89	12.17	0.06

**Figure 13:**
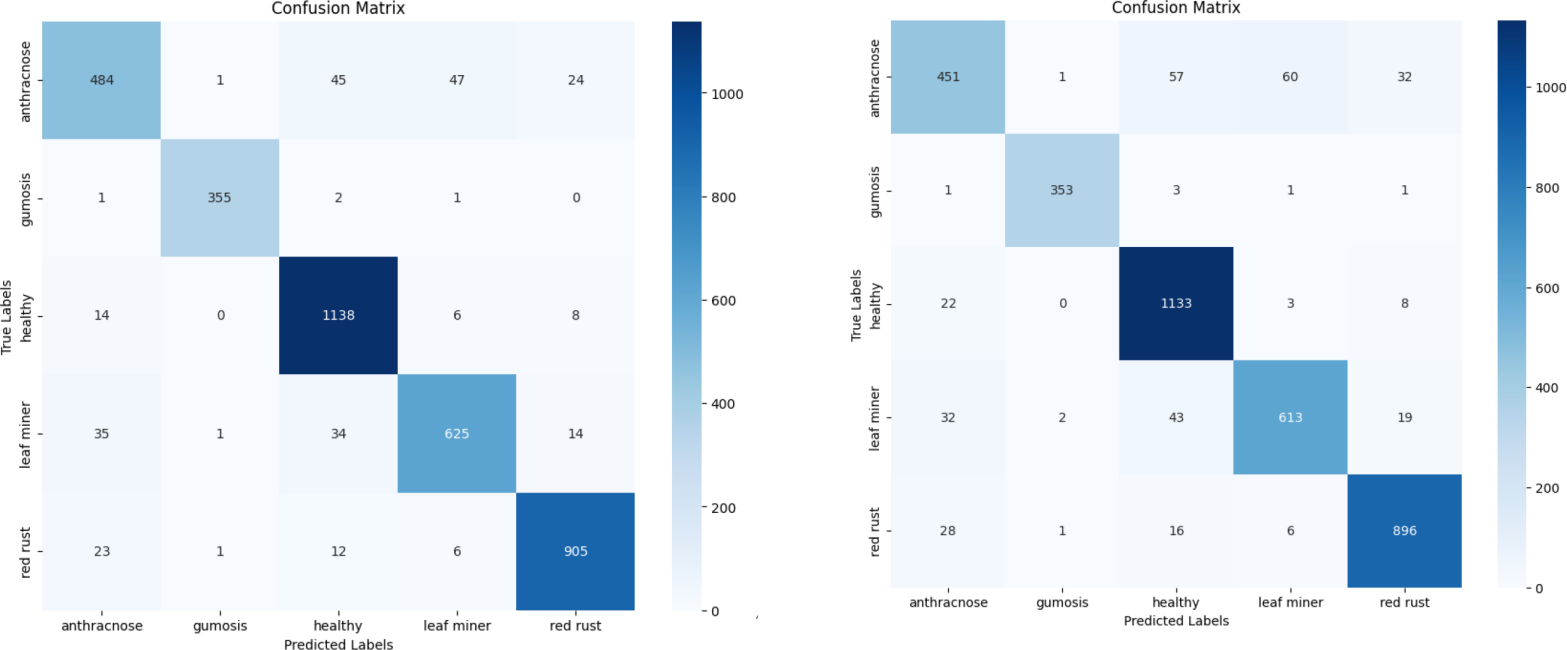
Confusion matrices – XGBoost and CatBoost classification with BOHB optimization.

To improve the accuracy of the proposed model, various optimisation techniques are used. Grid search optimisation and BOHB were used. The parameters are tuned based on the accuracy metrics. Totally 50 trials were executed to get the best parameters. At the end of the 40^th^ trial, we got the best values and it was tabulated in the **Table 6**. The accuracy of the proposed model with XGBoost classification with BOHB optimisation is 92.73 and its training time is 5.04 seconds. The confusion metrics of the XGBoost and Catboost models after tuning are displayed in **Figure 13**, where each branch generates a distinct set of features.

**Table 6:** BOHB optimisation – best parameter values XGBoost classifier.

S. no	Parameter	Initial value	Best value	Purpose
1	n_estimators	100-300 (step value 50)	250	number of boosting rounds (trees)
2	learning_rate	0.0001	0.1500	step size shrinkage, controls how quickly the model learns.
3	max_depth	3-20	4	maximum depth of trees, controls complexity.
4	Subsample	0.2-1.0	0.3643	fraction of training samples used per tree (helps avoid overfitting).
5	colsample_bytree	0.2-1.0	0.8603	fraction of features considered per tree (adds diversity).

**Figure 14:**
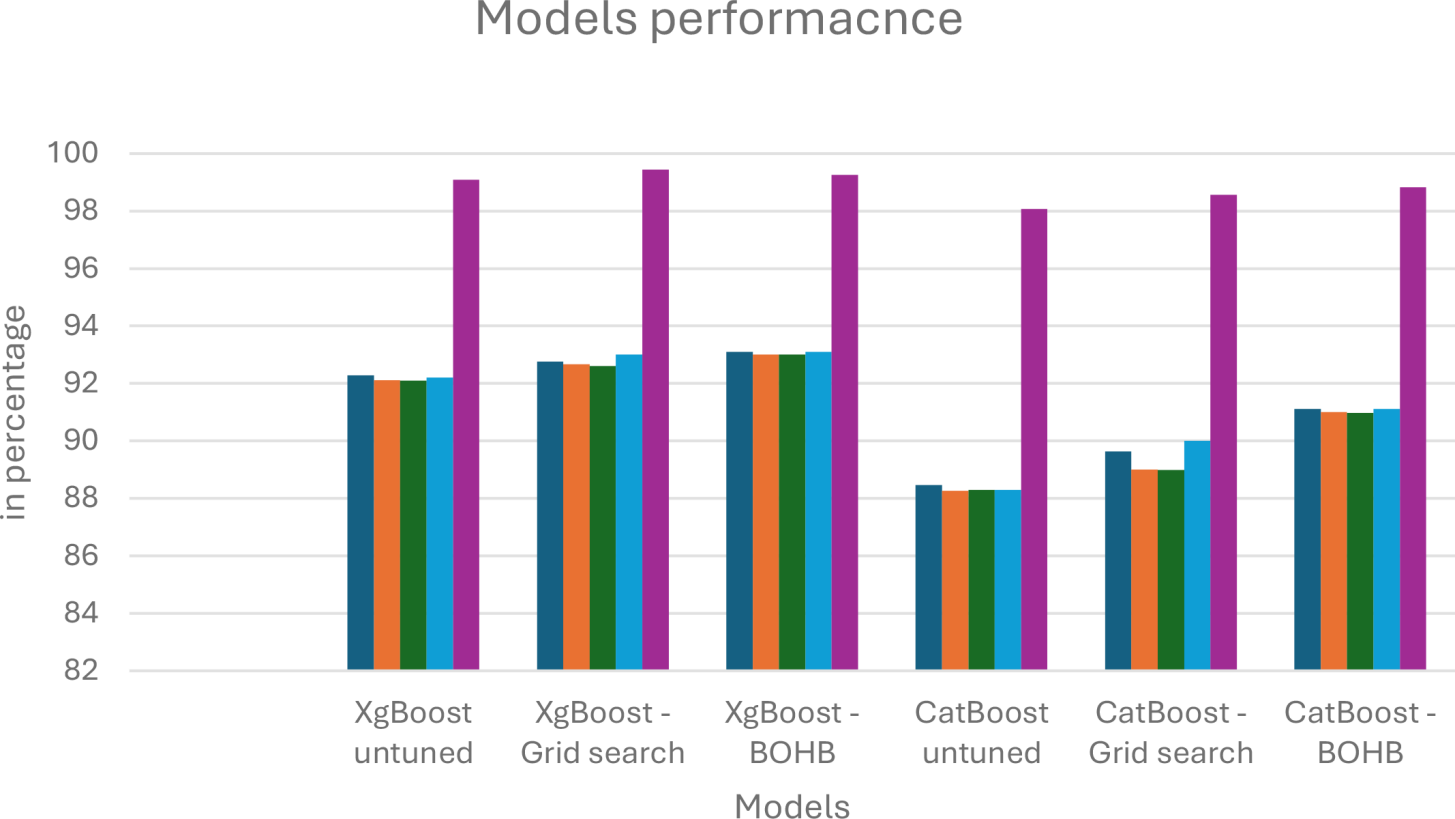
Comparison of proposed model – before and after parameters tuning.

**Figure 15:**
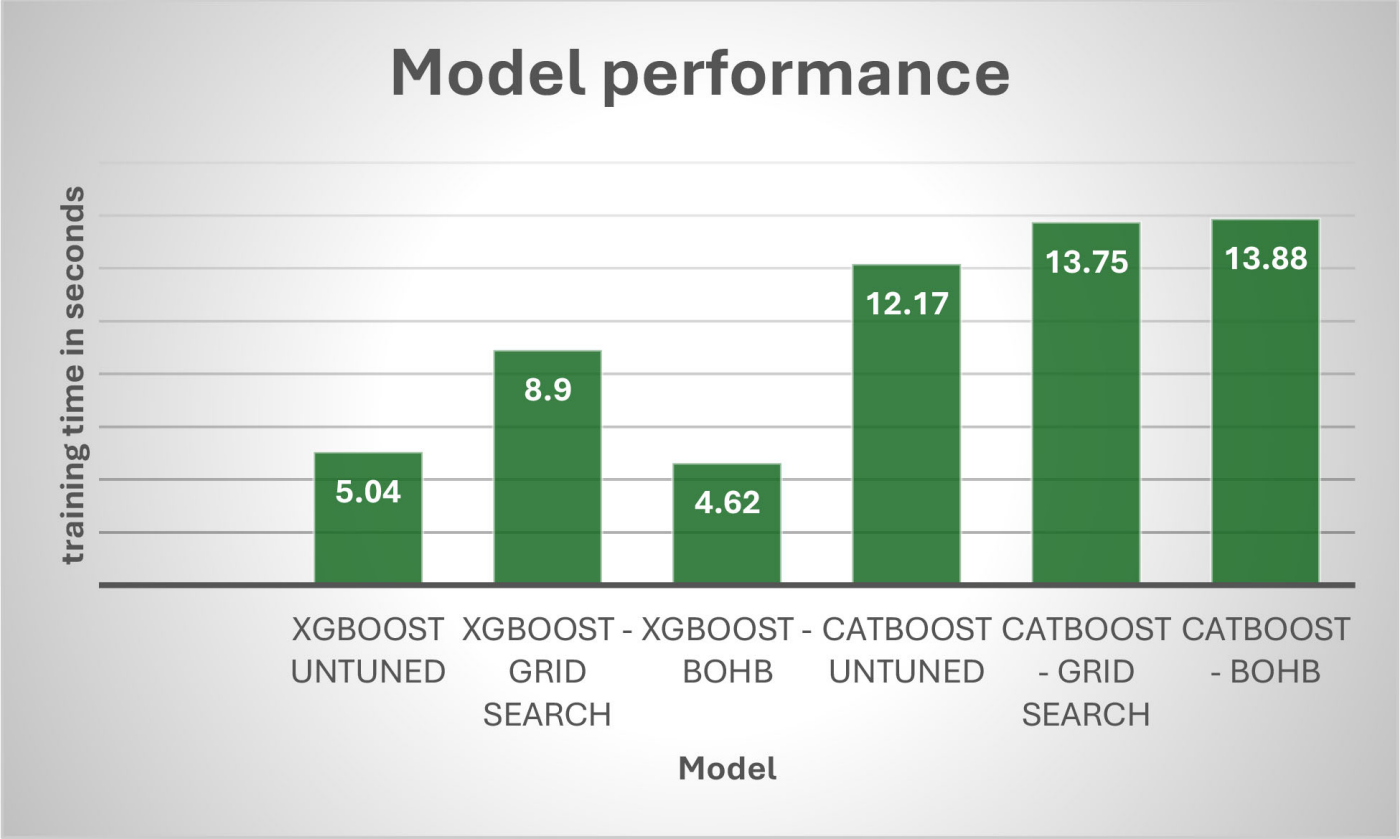
Performance of models – training time.

**Figure 16:**
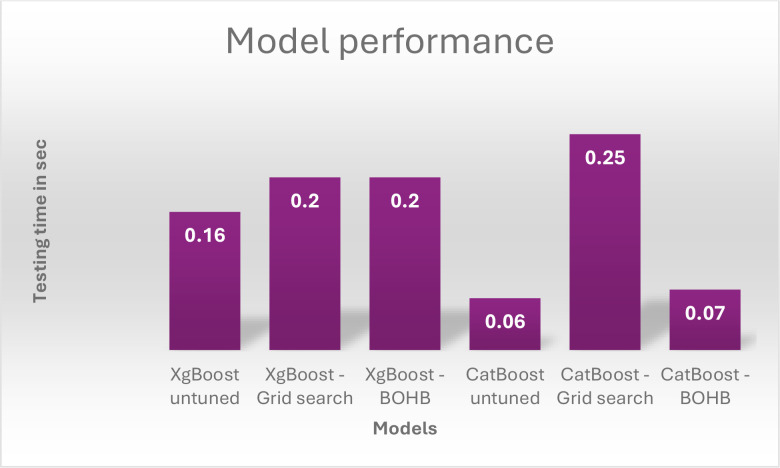
Performance of models – testing time.

The recall, precision, and f1-scores for the models used in the experiment are reviewed in **Table 7**. It presents the summary of performance metrics of the proposed feature-fused model with XGBoost and CatBoost classifiers. It also presents the optimisation results of Grid search and BOHB optimisation. From this, we can see that BOHB optimisation with XGBoost classification gives a good result with an accuracy of 93.10, precision of 93.01 and the training time is 4.62 seconds and evaluation time is 0.20 seconds. **Figures 14**–**16** presents the proposed model’s performance in terms of different metrics such as accuracy, recall, F1 score, precision, training time and testing time.

**Table 7:** Proposed model performance metrics.

Metrics/model	Train time (sec)	Accuracy	Precision	F1 score	TPR/recall	FPR	ROAUC	Test time (sec)
XgBoost untuned	5.04	92.28	92.11	92.10	92.20	2.04	99.09	0.16
XgBoost - Grid search	8.9	92.76	92.66	92.6	93	1.9	99.44	0.20
XgBoost - BOHB	4.62	93.10	93.01	93	93.10	1.8	99.26	0.20
CatBoost untuned	12.17	88.47	88.27	88.3	88.3	3.00	98.07	0.06
CatBoost - Grid search	13.75	89.64	89	88.98	90	3.05	98.56	0.25
CatBoost - BOHB	13.88	91.12	91	90.97	91.12	2.33	98.83	0.07

XgBoost generally outperforms CatBoost in classification metrics (Accuracy, Precision, F1, ROAUC) across all tuning strategies. For instance, the best CatBoost (BOHB) has an Accuracy of 91.12%, while the worst XgBoost (untuned) has 92.28%. Hyperparameter tuning significantly boosts performance for both algorithms. Comparing untuned to BOHB, XgBoost’s Accuracy improves from 92.28% to 93.10%, and CatBoost’s Accuracy improves from 88.47% to 91.12%. BOHB appears to be a more effective and efficient tuning strategy than Grid Search for these models: For XgBoost, BOHB yields better performance (e.g., Accuracy 93.10% vs. 92.76% for Grid Search) with a much shorter training time (4.62s vs. 8.9s). For CatBoost, BOHB also yields better performance (e.g., Accuracy 91.12% vs. 89.64% for Grid Search) while maintaining a significantly faster test time (0.07s vs. 0.25s for Grid Search). Test Time vs. Classification Accuracy: The unoptimized CatBoost and CatBoost-BOHB yield the shortest prediction times of 0.06 and 0.07 seconds, respectively. However, it must be noted that in practice, there can always be a trade-off between a very short prediction time and high classification accuracy. In contrast, the XgBoost-BOHB classifier demonstrates better classification performance at the cost of increased prediction time by almost twice (0.20 seconds).

In order to ensure statistical robustness, a performance evaluation of the proposed model was performed using the 5-fold cross-validation method. Specifically, each iteration involved training and testing the model independently and reporting its performance statistics, which are then averaged over five folds. The corresponding numerical values are presented in **Table 8**. As a result, the proposed hybrid model of XgBoost and BOHB achieves an average accuracy of 91.90% ± 0.0032. Consistent performance levels were also attained with regard to precision, recall, and F1-score.

**Table 8:** Models performance of repeated cross validation- parameters n_Split=5 scoring=‘accuracy’.

Metrics/model	Accuracy	Precision	F1 score	TPR/recall	ROAUC
Run 1					
XgBoost untuned	92.12	90.43	91.79	91.38	98.88
XgBoost - BOHB	92.17	92.33	91.79	91.43	98.90
CatBoost untuned	88.63	88.82	88.38	88.04	97.89
CatBoost - BOHB	90.93	91.28	90.52	90.01	98.53
Run 2					
XgBoost untuned	91.19	92.11	91.64	91.32	98.98
XgBoost - BOHB	91.99	92	91.66	91.39	99.4
CatBoost untuned	87.76	87.88	87.35	86.95	97.8
CatBoost - BOHB	90.72	91.19	90.40	89.8	98.66
Run -3					
XgBoost untuned	91.25	91.30	90.87	90.52	98.97
XgBoost - BOHB	91.33	91.35	90.93	90.57	98.97
CatBoost untuned	87.47	87.52	87.12	86.8	97.8
CatBoost - BOHB	90.03	90.24	89.68	89.27	98.72
Run 4					
XgBoost untuned	91.72	92.14	91.48	91.03	98.89
XgBoost - BOHB	91.80	92,04	91.59	91.22	98.92
CatBoost untuned	88.08	88.48	87.89	87.47	97.06
CatBoost - BOHB	90.69	91.27	90.55	90.05	98.6
Run -5					
XgBoost untuned	92.07	92.66	91.92	91.44	99.04
XgBoost - BOHB	92.23	92.8	92.03	91.48	99.05
CatBoost untuned	88.63	88.95	88.47	88.09	98.07
CatBoost - BOHB	90.72	91.28	90.44	89.87	98.72
Summary after repeated CV					
XgBoost untuned	Mean: 0.9183 ± Std: 0.0032	Mean: 0.9213 ± Std: 0.0046	Mean: 0.9154 ± Std: 0.0037	Mean: 0.9114 ± Std: 0.0034	Mean: 0.9895 ± Std: 0.0006
XgBoost - BOHB	Mean: 0.9190 ± Std: 0.0032	Mean: 0.9211 ± Std: 0.0047	Mean: 0.9160 ± Std: 0.0037	Mean: 0.9122 ± Std: 0.0034	Mean: 0.9898 ± Std: 0.0006
CatBoost untuned	Mean: 0.8811 ± Std: 0.0046	Mean: 0.8833 ± Std: 0.0055	Mean: 0.8784 ± Std: 0.0054	Mean: 0.8747 ± Std: 0.0053	Mean: 0.9790 ± Std: 0.0010
CatBoost - BOHB	Mean: 0.9062 ± Std: 0.0031	Mean: 0.9105 ± Std: 0.0041	Mean: 0.9032 ± Std: 0.0032	Mean: 0.8980 ± Std: 0.0028	Mean: 0.9865 ± Std: 0.0007

The experiments described in this section were carried out using an NVIDIA K80 GPU; nevertheless, it should be emphasized that the proposed approach is computationally efficient. Lightweight neural network architectures (MobileNetV3-S) and the decoupling of feature extraction and classification stages utilizing boosting models help reduce computational costs. Therefore, it can be predicted that the proposed model will perform even better when trained and evaluated on more advanced GPU architectures such as NVIDIA T4, V100, or A100. Moreover, the proposed model has great potential for deployment in precision agriculture using limited computing resources. Although the introduction of EfficientNetV2-M significantly increases the level of computational complexity, the use of MobileNetV3-S and the fusion of features allow for achieving a reasonable trade-off between accuracy and efficiency. Nevertheless, the performance of the proposed model under constrained computation conditions requires further research.

## 6 Benchmarking of results

A crucial benchmarking analysis is performed to compare the proposed EfficientNetv2M+MobilenetV3s with XGBoost (BOHB) with previous state-of-the-art solutions for plant and cashew leaf disease classification problem. Despite some solutions showing higher classification accuracies, careful analysis reveals several methodological drawbacks motivating this study. Jayaprakash et al (Foysal et al., 2024). reported an average accuracy of 98.0% using multiple Convolutional Neural Networks on a data-augmented cashew dataset. Nevertheless, their performance largely depends on heavy data augmentation and carefully selected dataset splits, potentially inflating the reported results and limiting the generalizability to real-world conditions. Besides, employing purely convolutional networks increases the complexity of the architecture, limiting its deployment on constrained systems. Foysal et al (Majumdar, 2025). obtained an accuracy of 98.14% in a very diverse problem setting by classifying a large dataset with 14 crops and 26 disease types. Despite obtaining such impressive results, using such a broad range of crops limits the specialization of the model in cashew leaf diseases. Also, building models with very complex architecture, such as the one used in this paper, requires significant training resources, preventing its use in cost-effective agricultural monitoring systems.

Majumdar et al (Jain and Nain). prove that combining XGBoost, a powerful boosting-based ensemble learner, with CNN features leads to high classification accuracies in some cases. Nevertheless, the performance varies significantly among different datasets, making this technique sensitive to hyperparameter configurations. Not using a systematic hyperparameter optimization technique also hinders reproducibility and scalability of results. Hybrid deep learning architectures have been developed to recognize specific diseases in cashews (https://www.kaggle.com/datasets/rahimanshu/ccmt-plant-disease-dataset), concatenate multiple neural networks in order to improve the overall performance. This type of architecture not only increases the complexity of the model, requiring higher computational power, but also makes overfitting even more common on moderate-sized agricultural datasets.

In order to perform a proper and rigorous comparison between proposed and other approaches, two additional sets of experiments were executed using (i) EfficientNetV2-M and (ii) MobileNetV3-S alone as classifiers with a Softmax activation function. Also, an experiment was performed using an hybrid network with a Softmax as output layer. Results are presented in **Table 9**.

**Table 9:** Benchmarking the result.

Ref.	Method (brief)	Dataset/scope	Classes (if reported)	Reported accuracy (%)	Notes
Jayaprakash et al (Jayaprakash and Rajagopal, 2023),	CNN variants (AlexNet/GoogleNet/MobileNet/ResNet) on a curated cashew dataset	Cashew dataset (augmented)	—	98.0	Dataset generation + augmentation; high reported accuracy on their split.
Foysal et al. (2024),	Multi-crop CNN model	Diverse multi-crop leaf dataset	14 crops/26 diseases	98.14	Large, diverse dataset — high overall accuracy reported.
Majumdar et al (Majumdar, 2025),	XGBoost on CNN features with Optuna tuning	Multiple plant leaf datasets	—	(reported competitive results; specific accuracies vary by dataset)	Demonstrates strong performance of tree ensembles when features are well engineered.
Hybrid Cashew Model (Jain and Nain),	Concatenated/hybrid deep models for cashew	Cashew leaf disease dataset	—	(reported competitive accuracies; dataset/split dependent)	Hybrid architectures designed specifically for cashew disease patterns.
EfficientNetV2M	Baseline Model	Cashew leaf dataset used in experiments	5 classes (Anthracnose, Gummosis, Healthy, Leaf Miner, Red Rust)	90.40 accuracy, training time of 1291.52 seconds and testing time of 52 seconds.	The baseline model takes high computation time in terms of training and testing.
MobileNetV3S	Baseline Model	Cashew leaf dataset used in experiments	5 classes (Anthracnose, Gummosis, Healthy, Leaf Miner, Red Rust)	91.90 accuracy, training time of 383 seconds and testing time of 37 seconds.	The baseline model takes high computation time in terms of training and testing.
Proposed hybrid late fusion model	EfficienetV2M+Mobilnet V3S (**Figure 8**) with XGBoost (BOHB)	Cashew leaf dataset used in experiments	5 classes (Anthracnose, Gummosis, Healthy, Leaf Miner, Red Rust)	93.10 accuracy, training time of 4.62 s and testing time of 0.2 seconds.	BOHB tuning produced best trade-off between accuracy and training time.

Unlike previous techniques, the Deep learning-XGBoost model is able to solve all of those shortcomings due to decoupling of the feature extraction and classification steps. Employing Bayesian optimization in combination with hyperbanding (BOHB) enables a systematic hyperparameter optimization with a good trade-off in terms of computational effort. Thus, our approach is able to obtain an accuracy of 93.10%, which is slightly worse than some other approaches, but more robust and practical.

## 7 Limitations of proposed work

Although the proposed model proves itself to be highly effective on the CCMT dataset, it should be noted that the experiments were carried out only using one specific dataset. Even though this dataset incorporates diversity in the aspects of illumination, complexity of background environment, and features of diseases, it cannot possibly provide variation as great as in a practical setting, which would span across different locations around the world. The ability of the model to generalize to unseen datasets still remains a task for future work, since further experiments on diverse sets of data have to be conducted.

## 8 Conclusion and future work

In this study, the strengths of deep learning and machine learning were effectively used to classify diseases and pests in cashew leaf diseases. This not only resulted in better performance in the models’ classification tasks but also reduced the need for computation. In this model, there were four classes of cashew leaf diseases being classified, namely, anthracnose, healthy leaf, leaf miner, and red rust. According to the results obtained from this research, the use of XGBoost classifier along with EfficientNet and MobileNet for deep features greatly increased classification accuracy. Moreover, the optimal parameters of the method can be found through BOHB optimisation. This method provides an accuracy rate of 93.10% while the time taken for evaluation is 0.20 seconds, which is superior to other related works. In addition, using a combination of deep learning models that have few parameters to perform feature extraction produces more reliable models than the ones with high parameters of CNNs.

Future works could expand upon this concept by including more diseases of cashew and other plants. Some improvements include the use of several data augmentations such as GAN models and different combinations of CNNs as feature extractors. Also, this research can be generalised to other problems with other feature extractors and other methods of fusion applied to various datasets. Finally, the model can be modified for application in mobile phones to help farmers detect and mitigate the problems of pests and diseases on their cashew crops in a timely manner.

## Data Availability

The original contributions presented in the study are included in the article/supplementary material. Further inquiries can be directed to the corresponding author.
